# Our Summer at Maplewood. Chap. VI—Breakfast Table Gossip

**Published:** 1875-07

**Authors:** 


### CHAPTER VI.

### A BREAKFAST TABLE GOSSIP.

"Kate, do I understand you to say that this coffee, which is so perfectly clear, has nothing used for settling it, but a spoonful of cold water." As I took the coffee she handed me, I answered, "At the time of making, nothing but water is added to the coffee; but at the time of browning, the white of one egg is mixed with a pound of coffee. Coffee can be settled perfectly with a spoonfull of cold water, but it stays settled better, and is not so easily disturbed or spoiled or roiled, where egg is used. A first rate cup of coffee, Emeline, is something rarely found on American tables, especially at the North, nor is it surprising, perhaps, when we consider that making coffee is a delicate matter, and that the beverage is easily spoiled, at any point from the beginning, to the end of the process. Many things are requisite in order to have good coffee. First, the coffee must be of good quality, carefully picked over, and cleaned by rubbing in a dry towel. Next it must be properly roasted. There are very perfect mechanisms for browning coffee, but any farmer's wife can roast it perfectly in the oven, if she attends to it. In a medium-sized dripping pan a pound can be roasted at a time. The person roasting the coffee should give it her undivided attention while it is in the oven, otherwise she will often forget, and the coffee will be spoiled. Coffee should roast slowly at first, an occasional shaking to change the position of the grains being all the attention it requires. After remaining ten or fifteen minutes in the oven, the heat may be increased, and the shaking must be done more frequently. Towards the last it must be looked at every minute or two, lest unawares it surprise you with too dark a browning. But how brown it should be depends somewhat upon the taste of the drinker and the kind of coffee used. Rio will bear a browner tinge than Java. If the coffee, when made, is pale in color, and tastes of the bean, that is, has a raw taste, it is not sufficiently roasted. If it is black and tastes bitter and burnt, it is overdone. It is a good plan to keep a spoonful of it unground, from one roasting till the next, in order to compare the newly roasted with it. When the coffee is roasted, pour it into an earthen dish to cool; and when so cool that you can clasp a handful of it tightly in your palm and feel it only warm—not hot—add to it the white of a fresh egg, and with a fork, stir it until perfectly mixed and each kernel has a share of the egg. The warmth of the coffee should be just sufficient to dry the egg adhering to it, and not cook it. When dry, the egg does not interfere with the grinding. Egg used in this manner is neither so troublesome nor expensive as when added at the time of making the coffee. Another convenient, inexpensive method is, to wash eggs before breaking, and preserve the shells for settling coffee.

The grinding of coffee is an important consideration. If ground very coarse, much of the strength remains in the grounds, some of which are often seen floating about in your cup in an independent manner, and an isolated condition. If ground too fine it will be muddy in spite of everything. Of course a happy medium is right. Some ladies have coffee prepared in one pot and poured into another before sending to the table; but this is a bad practice, for every time coffee is exposed to the air it loses some of its aroma and excellence. When I have a fragrant perfume of coffee in my chamber while dressing, I take it for granted that I shall miss at breakfast the delicious aroma that has been so freely exhaled all over the house. If it is borne to me on the wings of the morning up three flights of stairs how can I hope to find it concentrated in my cup at breakfast? Really excellent coffee can be made in various ways. The most perfect method of making it, perhaps, is by dripping the boiling water through the coffee. But rare coffee may be made in the ordinary manner of steeping, if it is not allowed to boil enough to extract the bitter, or waste the aroma. There is one thing you must insist upon, cousin Emeline, if you would have the best quality of coffee and tea, namely, *the pots must be kept clean on the inside.* If carefully washed every time they are used, they will not become dark and coated with deposits. Coffee made very strong and diluted with boiled milk instead of water, is liked by many. That is a French style worthy of imitation. Mixed coffees are also considered by many finer in flavor than a single sort. I have tried various combinations, and like Rio and Java in equal proportions—or two parts Rio, two parts Java and one part Mocha. To be most perfect, I think the different kinds should be roasted separately, and mixed afterward. But my advice to housekeepers is, experiment. Find out for yourselves what is best, and don't believe implicitly in any cook-book, not even Cousin Kate's. Use cook-books as helps, as hints, but not as infallible guides, and work out your own salvation in cookery by the use of your own brains. A week's supply of coffee may be browned and ground at one time if it is kept in air tight cans or canisters."

While we were loitering over breakfast, discussing the merits of coffee, Tom, the gardener's son, brought us the morning mail. Among various and sundry letters, postal cards, etc., I found the following missives, the first from an old bachelor cousin of mine, and the other from an intimate lady friend:—

MY DEAR KATE:

You have given such minute and admirable directions for bread-making that it seems to me no woman of sense who studies them carefully, and follows them faithfully can fail to have bread of the very best quality. And I hope you will speedily tell the readers of HALL'S JOURNAL how to make good tea. For though it would seem at first blush that any one could make tea, it is a singular fact that at the average table good tea is as great a rarity as good bread. I confess to a weakness for 'the cup that cheers but not inebriates,' for to my taste no other beverage is so delicious as well made tea; but for the sloppy, bitter or smoky tasting decoctions that are foisted upon us at hotels, restaurants, and private houses under that name, I have the most ineffable contempt. It is an insult to any decent stomach to force into it the wretched slops so barbarously extracted from theChinese herb. Make haste, my dear Kate, to enliven the world upon this subject. Explain how the tea is made that we occasionally find, and the fragrant memory of whose delicious flavor lingers with us forever after,effectually spoiling our relish of the common, vile decoction. By so doing you will earn the everlasting gratitude of

COUSIN JOHN.

As I finished reading the letter,Emeline observed, " I think Cousin John would be quite satisfied with the English breakfast tea you gave us here, Kate; for I never drank finer, even in England. How long do you boil it, and how do you manage always to have it just right?" " I do not boil English breakfast tea at all. And following precisely the same method of making every time, why should the quality of the tea vary? I think it would be safer for a careless, inattentive cook to boil the tea, if it did not boil more than a minute, for to make it without boiling, three requisites must be faithfully observed. The pot must be as hot, when the tea is put into it, as boiling water can make it. The water must be boiling when poured upon the tea, and the tea must stand, before being poured, from five to ten minutes. This is the receipt I shall give for making tea for Cousin John. Ten or fifteen minutes before making the tea, fill the teapot with boiling water, and the tea kettle with cold water, freshly drawn. It makes a vast difference with the quality of the tea whether it is made of water freshly boiled or of stale water that has stood in the kettle and been re-boiled, until all the sparkle and life have gone out of it. As soon as as the kettle boils, empty the tea pot, rinsing it with boiling water. Put into it a heaping teaspoonful of tea to each person, allowing a cup and a half of water to the same. Cover closely, letting it stand from five to ten minutes before pouring. Cream is as essential to good tea as it is to good coffee; and in both cases is best when the hot liquid is poured upon it. When the tea is all poured from the pot, boiling water may be added to extract the remaining strength from the leaves."

Fanny's letter ran thus: May all good spirits speed you, dear Kate, in your efforts to ameliorate the unhappy condition of the people who eat. Unfortunately I'm in a position to appreciate the need of your labors, for alas! I'm boarding. For dinner today we had spring lamb and green peas, the first of the season, and both were unfit to eat by reason of being under done, that is, raw. After tasting the peas I shoved them away in disgust, and with an injured feeling which I think not conducive to digestion, I picked out bits of lamb so far cooked that the squeaking sound it made while undergoing friction from my teeth, only in a very faint degree, resembled the far off bleating of a lamb; and that resemblance, I'm certain, was pure imagination, for I have no doubt the lamb was dead even if it was not cooked. Had the peas been sufficiently boiled, they would still have been nearly worthless, as our cook belongs to that class of idiots who drain peas; deliberately pour away their life and sweetness, leaving for our nourishment only the dry husks. I hope in your book you will castigate such cooks severely Another senseless practice is to boil peas in a bag in a pot of water. The only advantage this method has, is that soup is sometimes made of the water—which is a very questionable one; for to eat soup in warm weather, in order to save the juice of the peas is like the economy of taking medicine to prevent its being wasted. I doubt if *you* can give a better receipt for cooking green peas than this: To a quart of geen peas add a half pint of boiling water, cover closely, and simmer gently until very tender, at which time the water should be nearly evaporated. Season *lightly* with salt and pepper, adding a little butter, or sweet cream. I wish you to italicize that word *lightly*; for most cooks kill peas with salt. But please suggest that good taste requires that peas should be moist enough to necessitate being served in separate dishes. I detest peas so hard and dry that they roll and tumble about my plate, when I make vain efforts to entice them on my fork, and I usually soon abandoned the fruitless chase. And, Kate, don't forget the claims of asparagus. For next to peas, I think it suffers most at the hands of stupid cooks. I have seen it served at all the intermediate stages between aspargus soup, and asparagus mush; and have known it to be mixed with dandelions, mustard, and lamb's quarter. In rinsing, it is not enough to pour water over the asparagus. Each stalk should be held separately by the base and swashed back and forth in a large pan of water until free from sand and dust. Then it should be tied in small bunches, and laid carefully into a stewpan of boiling water, slightly salted. Let the water barely cover the vegetable. Simmer gently until very tender, and perfectly cooked. Have ready a platter covered with small slices of dry toast. With a fork lift a bunch of asparagus by means of the string around it, let it drip for an instant, then place it carefully upon a slice of toast. When all is so placed, cut and remove the strings. Pour over it drawn butter made with milk, or simply melted butter. The toast is not only a palatable accompaniment, but is useful in saving the vegetable. Yours in haste,

FANNY.

I known of no better method of cooking peas or asparagus than those described by Fanny, and shall certainly preserve her letter among my cook book scraps." Emeline added a postscript, as it were, to Fanny's letter, by saying, "Kate *I* beseech you in behalf of the potato—the white potato, which we eat in season and out of season every day of our lives. What a sorry, sad looking object it is as it comes from the hands of some cooks, sent to the table in its dingy jacket, or sallow and waxen without its jacket, that having been hastily removed just before serving. But served in either of these styles it is preferable to that miserable mash, bluish in color, full of lumps and cold stiffness, or watery and rank in flavor. Your descriptions are perfect, Emeline, I recognize each as an old acquaintance. The white, or Irish, potato when full grown, I think, is always better pared before cooking. Not only pared, but well pared. Having carefully removed all the dark eyes and dingy spots, let the potatoes lie for an hour or two in a plentiful supply of cold water, and then put them to boil, in a large quantity of boiling water. They should boil moderately, and as soon as tender at the heart, be drained, partly covered and set to dry on the back of the stove. Every minute or two give them a gentle shake, so that they roll over and change positions, until they are perfect snowballs of powdery whiteness. If served in this state, you will find them the perfection of boiled potatoes. I think very few would pass them by untasted. If mashed, a pestle should be used for the purpose, and the potato made fine and free from all lumps, before cream, milk, or butter is added. After the seasoning is in, beat well with a wooden spoon, or potato-beater, until light. Serve hot. An agreable companion to fried chicken is fried potato done in this way : Slice very thin, soak for an hour or two in cold water, changing the water once or twice. Drain the slices in a strainer, and dry them on towels, by rolling and tumbling them from one towel to another. Have the slices separate, not packed together, when put into the boiling lard. Fry the same as chicken. When of a light brown, lift, by means of a wire skimmer, (an indispensible thing in every well ordered kitchen), and drain in a wire or tin sieve, in a warm oven, until served. Serve hot. The last water in which the slices lie may be slightly salted, or a very light sprinkling of salt given them when served. Stewed potato, a nice breakfast dish, is easily prepared. Slice the potato much thinner than for frying, boil gently until tender, drain the water away, and add sweet cream, or milk and seasoning. String beans and lima beans should be cooked in the same manner as green peas. Dried beans, a palatable nutrition vegetable, is seldom seen upon our tables, save as the old time-honored traditional, baked pork and beans, a dish which the average modern stomach rejects as too strong. But why discard the bean because the discovery of trichina has spoiled our relish for pork ? To cook dried beans place them on the back of the stove in a plentiful supply of cold water. When swelled, drain the water off and give them a fresh supply. Let them simmer until parboiled. Drain again adding enough hot water to finish cooking. Boil gently till soft, and when done, season with salt, pepper and butter. Serve them at this stage as stewed beans ; or place them in a deep dish, and bake for half an hour in a hot oven, and you have baked beans, a palatable dish, without the evil spirit of pork, disfiguring its fair proportion. Butremember, in cooking beans for human stomachs, they cannot by any possibility be cooked too much. When done and served not a bean should be visible. Whole beans rarely digest, and imperfectly cooked beans are about as uselss as bullets, as an article of food, while nothing can be more nutritious than beans thoroughly done."

